# Estimation of gait events and kinetic waveforms with wearable sensors and machine learning when running in an unconstrained environment

**DOI:** 10.1038/s41598-023-29314-4

**Published:** 2023-02-09

**Authors:** Seth R. Donahue, Michael E. Hahn

**Affiliations:** Department of Human Physiology, Bowerman Sports Science Center, University of Oregon, Eugene, OR 97403 USA

**Keywords:** Mechanical engineering, Applied mathematics, Computer science, Scientific data

## Abstract

Wearable sensors and machine learning algorithms are becoming a viable alternative for biomechanical analysis outside of the laboratory. The purpose of this work was to estimate gait events from inertial measurement units (IMUs) and utilize machine learning for the estimation of ground reaction force (GRF) waveforms. Sixteen healthy runners were recruited for this study, with varied running experience. Force sensing insoles were used to measure normal foot-shoe forces, providing a proxy for vertical GRF and a standard for the identification of gait events. Three IMUs were mounted on each participant, two bilaterally on the dorsal aspect of each foot and one clipped to the back of each participant’s waistband, approximating their sacrum. Participants also wore a GPS watch to record elevation and velocity. A Bidirectional Long Short Term Memory Network (BD-LSTM) was used to estimate GRF waveforms from inertial waveforms. Gait event estimation from both IMU data and machine learning algorithms led to accurate estimations of contact time. The GRF magnitudes were generally underestimated by the machine learning algorithm when presented with data from a novel participant, especially at faster running speeds. This work demonstrated that estimation of GRF waveforms is feasible across a range of running velocities and at different grades in an uncontrolled environment.

## Introduction

Biomechanical analysis of running outside the laboratory has become possible, due to advances in wearable sensor and machine learning technologies^1,2^. Laboratory based technologies such as motion capture and instrumented force plates have been the traditional method with which to measure biomechanical data, including spatial–temporal, kinematic and kinetic variables. These laboratory-based tools require significant investment and high levels of training to collect, process and analyze these data. Wearable technologies are an alternative to laboratory based methods and have become more widely available for the monitoring of running biomechanics in uncontrolled environments^3,4^. Examples of these are inertial measurement units (IMUs), GPS, and in-shoe force or pressure sensors, which can be used to estimate or measure biomechanical data^5–8^. Earlier research has utilized IMUs for the estimation of gait events and foot ground contact time both in and out of the laboratory^5,9–13^. Estimation of specific kinetic variables with statistical or machine learning models has been completed strictly in the laboratory^14–16^. In-shoe force sensors measuring normal force between the foot and shoe during foot contact have been validated as a measure of vertical ground reaction forces (GRFs) on an instrumented force treadmill, which allows for basic kinetic analysis to be completed outside of the laboratory^7^.

There are typically 9 sensors in a commercially available multi-axial IMU: tri-axial accelerometers (linear accelerations), tri-axial rate gyroscopes (angular velocity), and tri-axial magnetometers (magnetic field). Data from IMUs need specific processing and algorithms for extraction of meaningful biomechanical variables^17^. Some approaches have been developed specifically for running, with sensors located on the foot, shank, and sacrum^9,10,18,19^. These algorithmic techniques have demonstrated consistent features can be extracted from inertial data for identification of foot contacts in the laboratory and in real-world environments. However, these algorithms are yet to be validated against a kinetic measure in a free running real-world environment, with uncontrolled running velocities and different positive and negative grades.

Machine learning models have been implemented for estimation and prediction of gait events^20,21^, of single kinetic variables^14–16^, and single stance phase GRFs during running^15,22–24^. These studies have been constrained to the laboratory, with either in-ground force plates or instrumented force treadmills. While there have been numerous approaches, and models used, it seems that an optimal machine learning model for the estimation of GRF waveforms are Long Short-Term Memory networks (LSTMs) and Bi-Directional LSTMs (BD-LSTMs). These network structures were designed for the analysis of temporally related data, specifically natural language processing^25^. Human gait data are ideal for these types of algorithms, as locomotion is cyclic. However, we must be cautious with the application of machine learning algorithms trained on data collected in the laboratory for evaluation of running performance outside of the laboratory, as it has been well established that gait parameters, kinematics and kinetics are different between treadmill running and overground running of different durations^26–30^. It is currently unknown how fully data driven models with no feature engineering performs with data collected over the course of an entire run over different grades and velocities.

The purpose of this study was to test two specific methods for the biomechanical analysis of running in an unconstrained environment: (1) a heuristic algorithm for the estimation of foot contacts from IMU data; (2) a machine learning algorithm with no feature engineering, Bi-Directional LSTM (BD-LSTM), for estimation of normal GRFs between the foot and shoe; and therefore, the estimation of gait events and calculation of discrete GRF variables. We expect gait event detection from both algorithms to have similar accuracies across the range of running velocities and grades in this study. Specifically, we expect a Root Mean Squared Error (RMSE) of 0.04 s, or 6% error, in the estimation of foot contact from the IMU data, which is similar to the results reported by Benson et al.^5^. Finally, we expect that estimated stance phases (assessed from waveforms output from the machine learning algorithm), would have an RMSE of 0.030 BW, and estimated discrete kinetic variables would have moderate correlations with measured variables, similar to previous work^22^.

## Results

There were 90,537 foot strikes measured with the force sensing insoles. Algorithmic output data from the foot mounted IMU heuristic estimated a total of 90,063 (88,364 analyzed) foot strikes, and the BD-LSTM estimated 90,579 (85,406 analyzed) foot strikes. The average pace and running speed are shown in Table 1. Two participants ran different courses than initially prescribed, each longer than 5 miles. Data from all folds of the LOOCV are presented in Table 1.

**Table 1:** Participant characteristics.

Sex	Age	Mass (Kg)	Height (cm)	Steps Measured	Average Speed (m s^−1^)	Pace (mins:secs)
M	19	68	175	10,209	3.62	7:24
F	21	63	168	7215	3.35	7:59
M	21	73	183	6094	3.67	7:15
F	19	55	173	5810	3.62	7:24
M	34	68	185	6407	3.23	8:17
F	23	52	163	6888	3.39	7:51
M	18	68	183	6317	3.60	7:33
M	20	68	170	6434	3.65	7:13
F	27	54	173	8103	3.01	8:57
M	28	88	191	7711	2.93	9:10
M	26	70	169	6787	3.72	7:13
F	27	57	165	7131	3.16	8:31
M	18	61	183	6358	3.16	8:31

**Figure 1:**
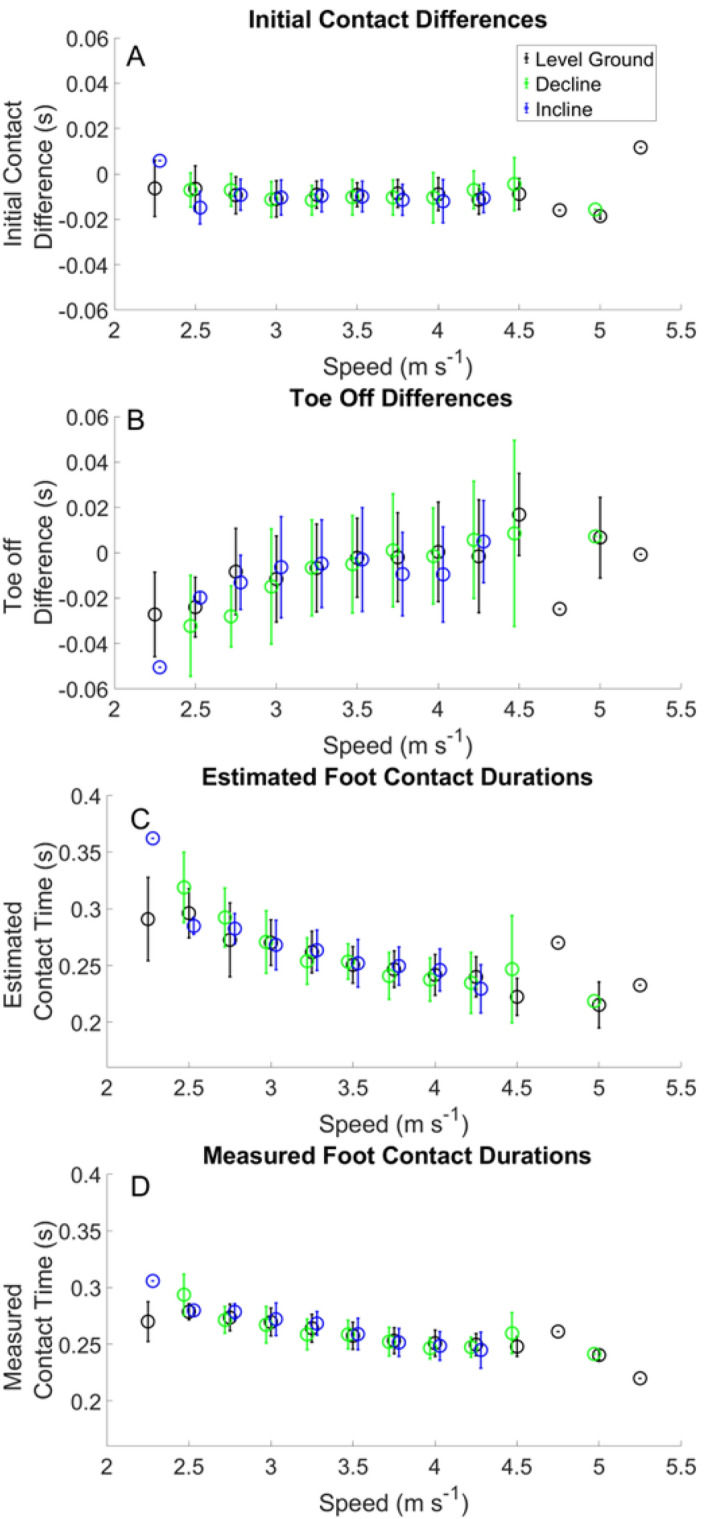
Gait event differences estimated from foot mounted IMUs (Panels **A** and **B**). Panels (**C**) and (**D**) show the estimated and measured contact times.

**Figure 2:**
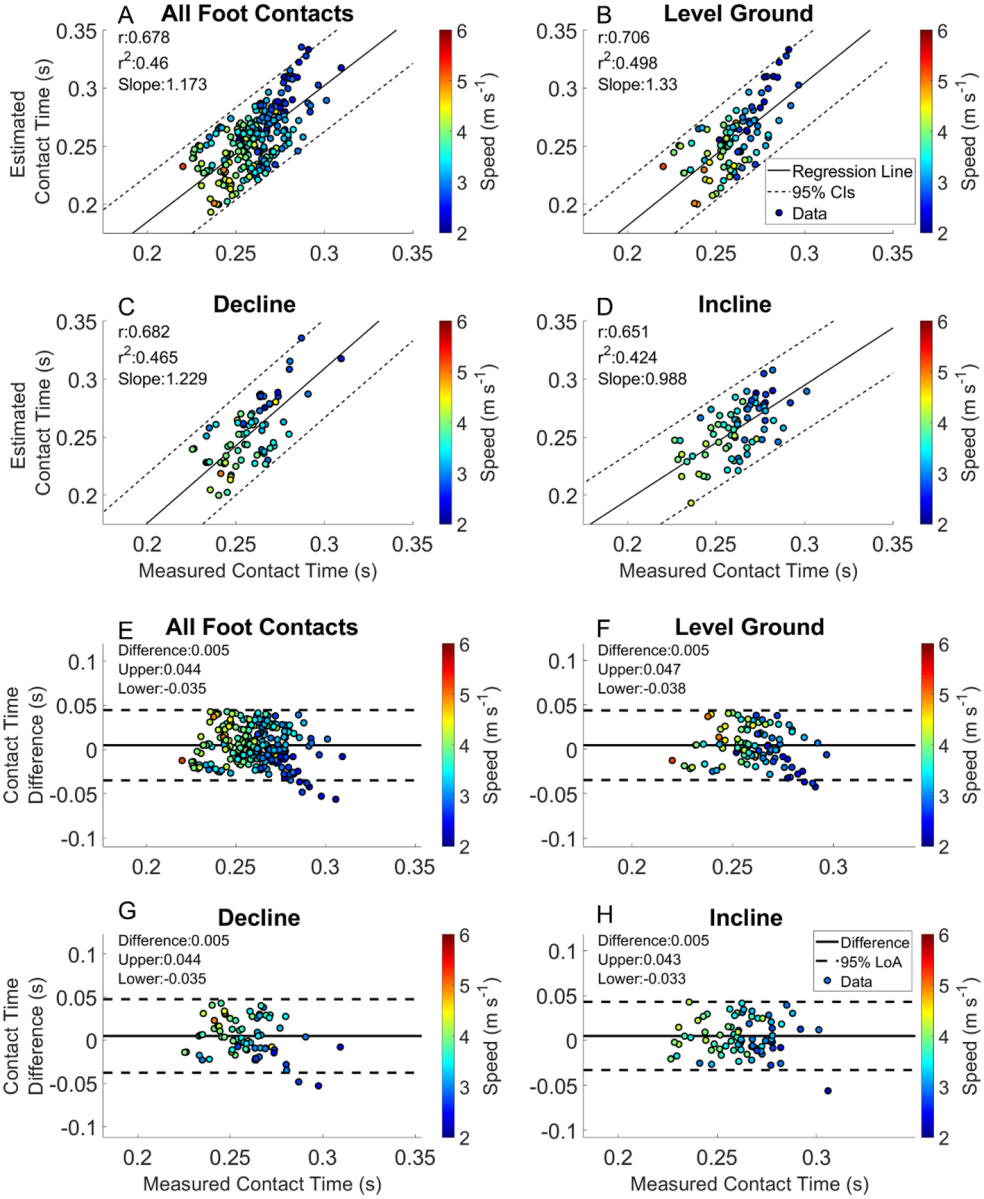
Estimated contact time from foot mounted IMUs. Linear regression and Bland–Altman plots are presented for all foot contacts (**A** and **E**), followed by level ground (**B** and **F**), decline (**C** and **G**) and incline foot contacts (**D** and **H**). Pearson Correlation Coefficients, and the slope of the regression line are presented in panels (**A**)–(**D**). The Bland–Altman plots present differences between the estimated and measured contact time. The average difference and the 95% LoA are shown in panels (**E**)–(**H**).

**Figure 3:**
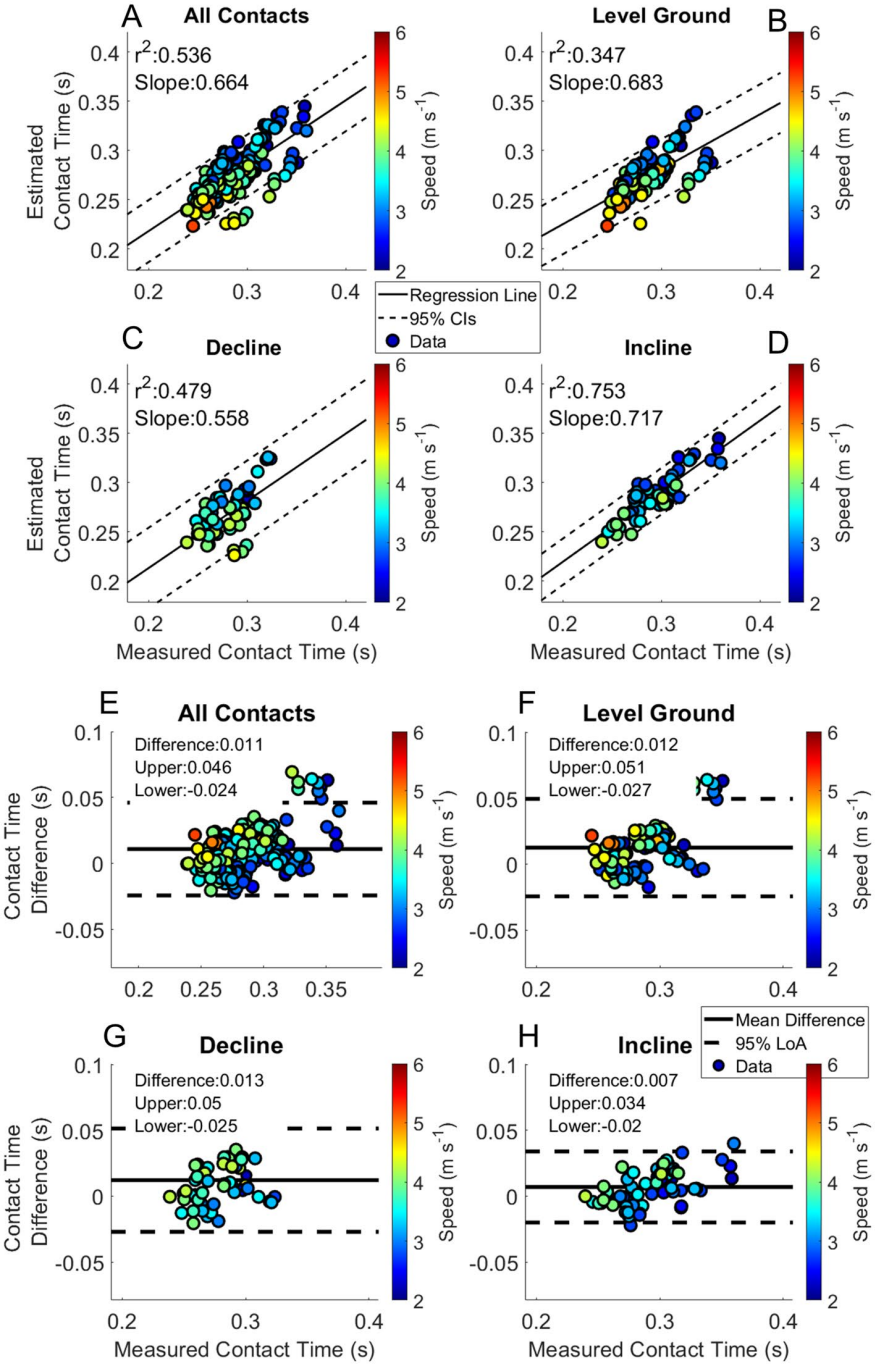
Estimated contact time from the BD-LSTM. Linear regression and Bland–Altman plots are presented for all foot contacts (**A** and **E**), followed by level ground (**B** and **F**), decline (**C** and **G**) and incline foot contacts (**D** and **H**). Pearson Correlation Coefficients, and the slope of the regression line are presented in panels (**A**–**D**). The Bland–Altman plots present differences between the estimated and measured contact time. The average difference and the 95% LoA are shown in panels (**E**–**H**).

**Figure 4:**
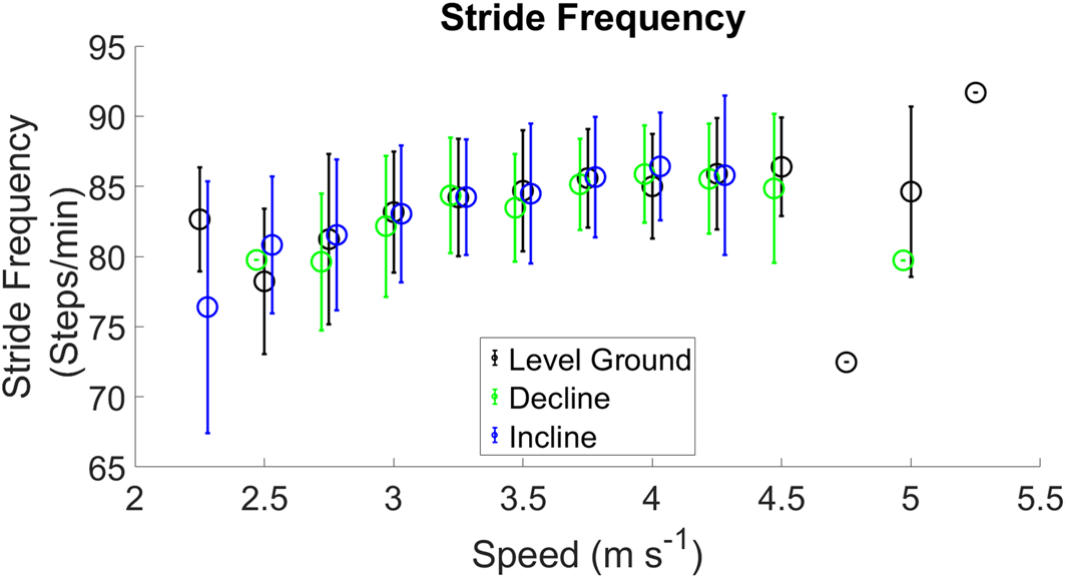
Stride Frequency measured from the force sensing insoles, across the range of velocities and grades.

**Figure 5:**
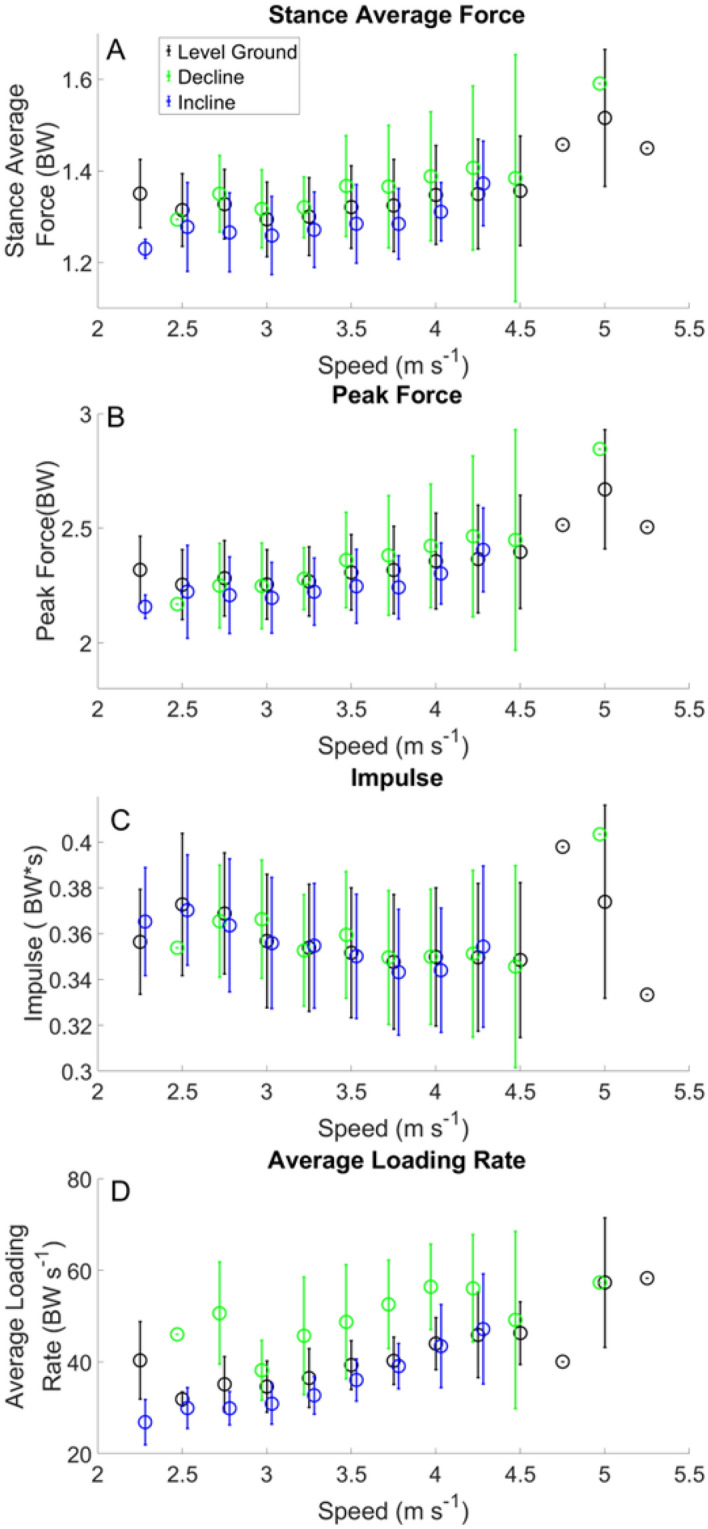
Kinetic variables across the range of running velocities and grades.

**Figure 6:**
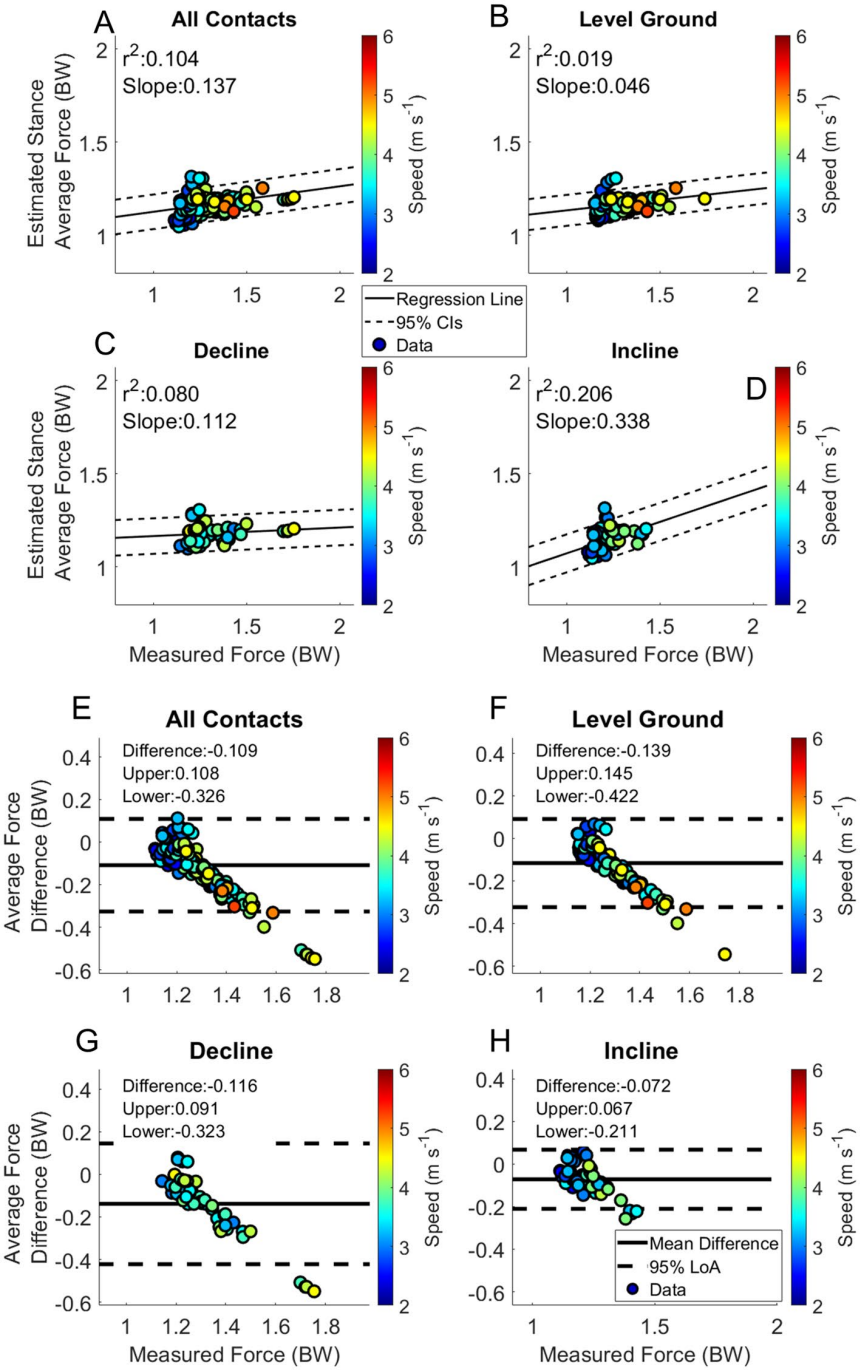
Stance average GRF. Linear regression and Bland–Altman plots are presented for all foot contacts (**A** and **E**), followed by level ground (**B** and **F**), decline (**C** and **G**) and incline foot contacts (**D** and **H**). Pearson Correlation Coefficients and the slope of the regression line are presented in panels (**A**–**D**). The Bland–Altman plots present differences between the estimated and measured stance average GRF. The average difference and the 95% LoA are shown in panels (**E**–**H**).

**Figure 7:**
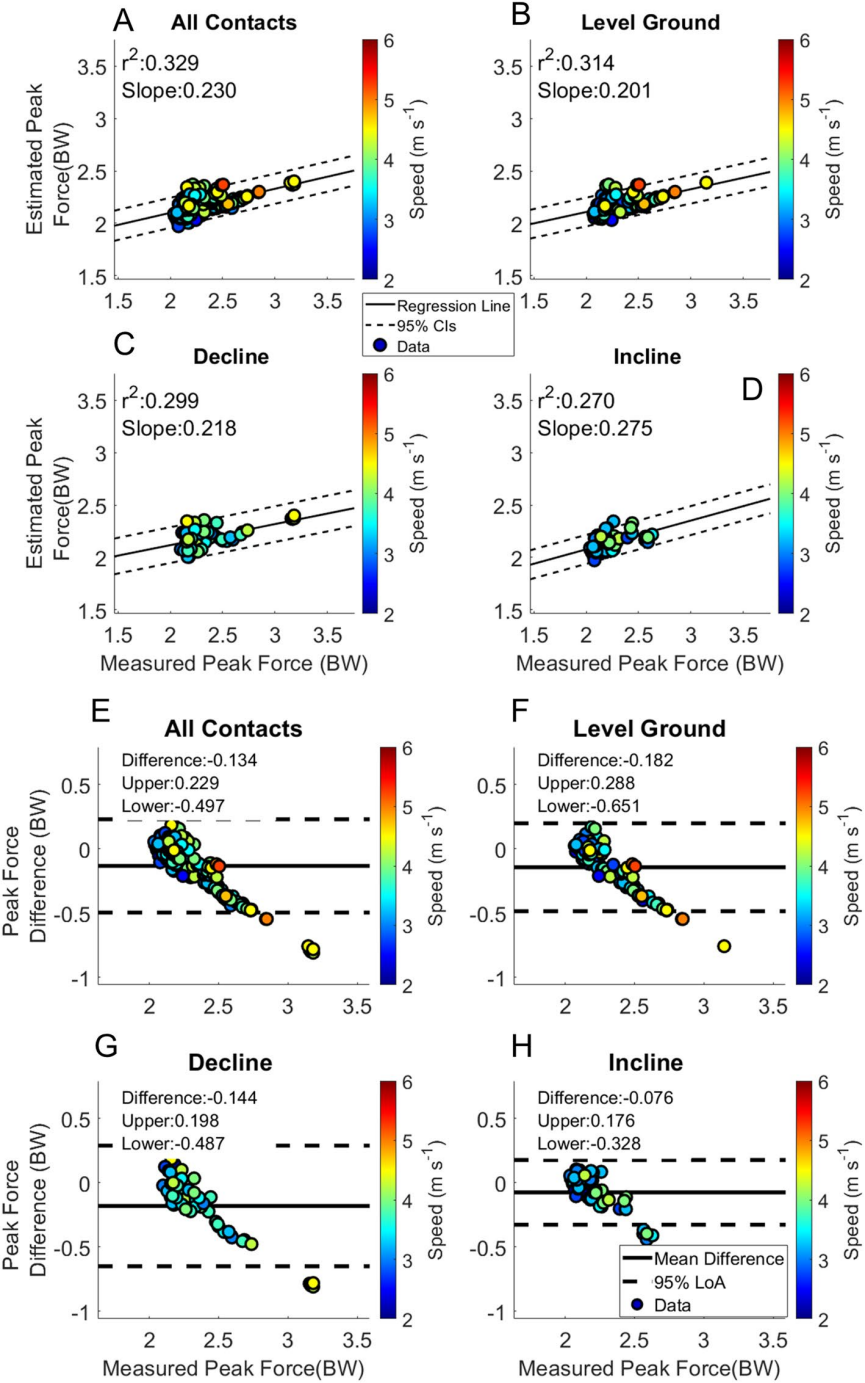
Peak Force. Linear regression and Bland–Altman plots are presented for all foot contacts (**A** and **E**), followed by level ground (**B** and **F**), decline (**C** and **G**) and incline foot contacts (**D** and **H**). Pearson Correlation Coefficients and the slope of the regression line are presented in panels (**A**–**D**). The Bland–Altman plots present differences between the estimated and measured stance average GRF. The average difference and the 95% LoA are shown in panels (**E**–**H**).

**Figure 8:**
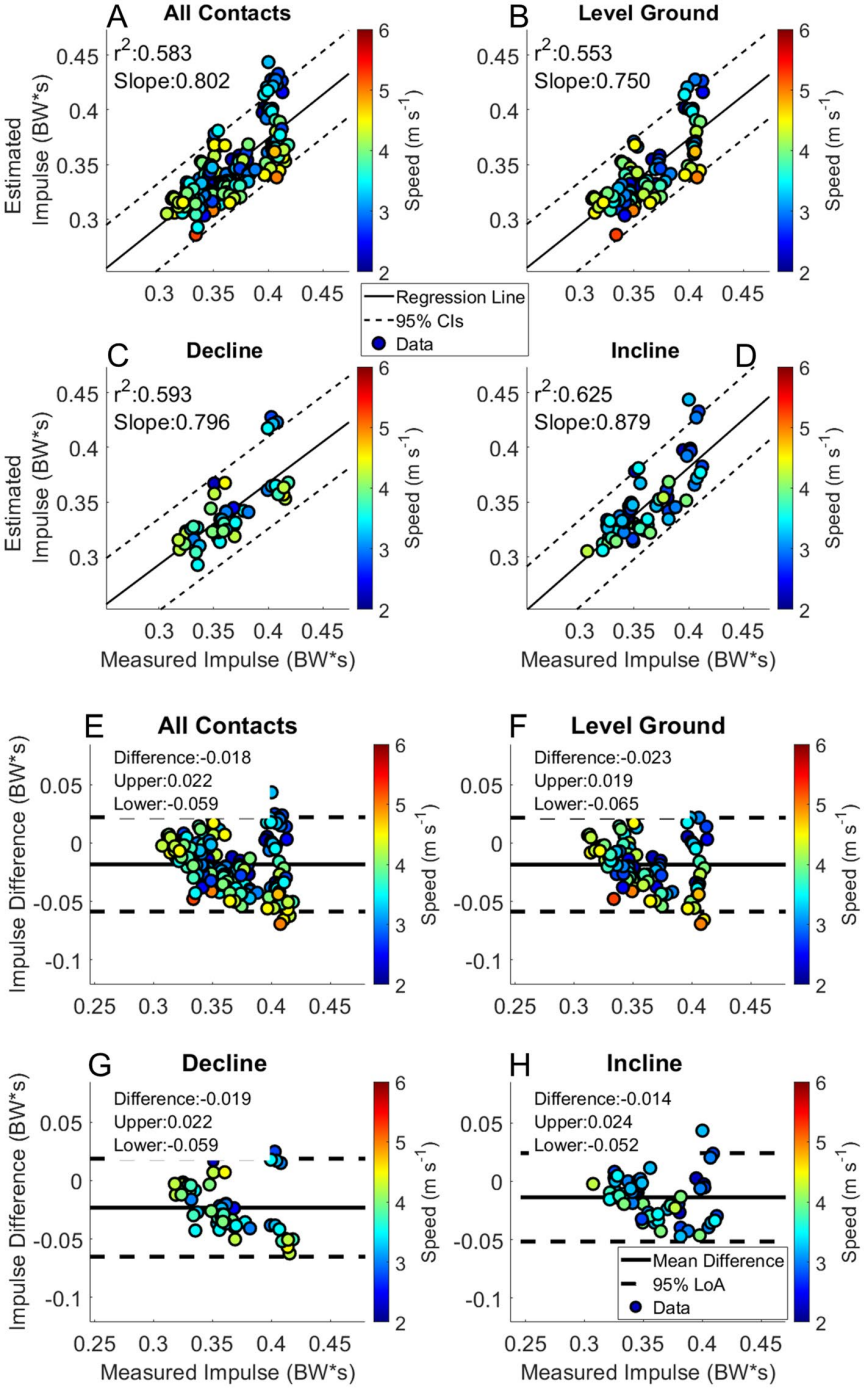
Impulse. Linear regression and Bland–Altman plots are presented for all foot contacts (**A** and **E**), followed by level ground (**B** and **F**), decline (**C** and **G**) and incline foot contacts (**D** and **H**). Pearson Correlation Coefficients and the slope of the regression line are presented in panels (**A**–**D**). The Bland–Altman plots present differences between the estimated and measured stance average GRF. The average difference and the 95% LoA are shown in panels (**E**–**H**).

**Figure 9:**
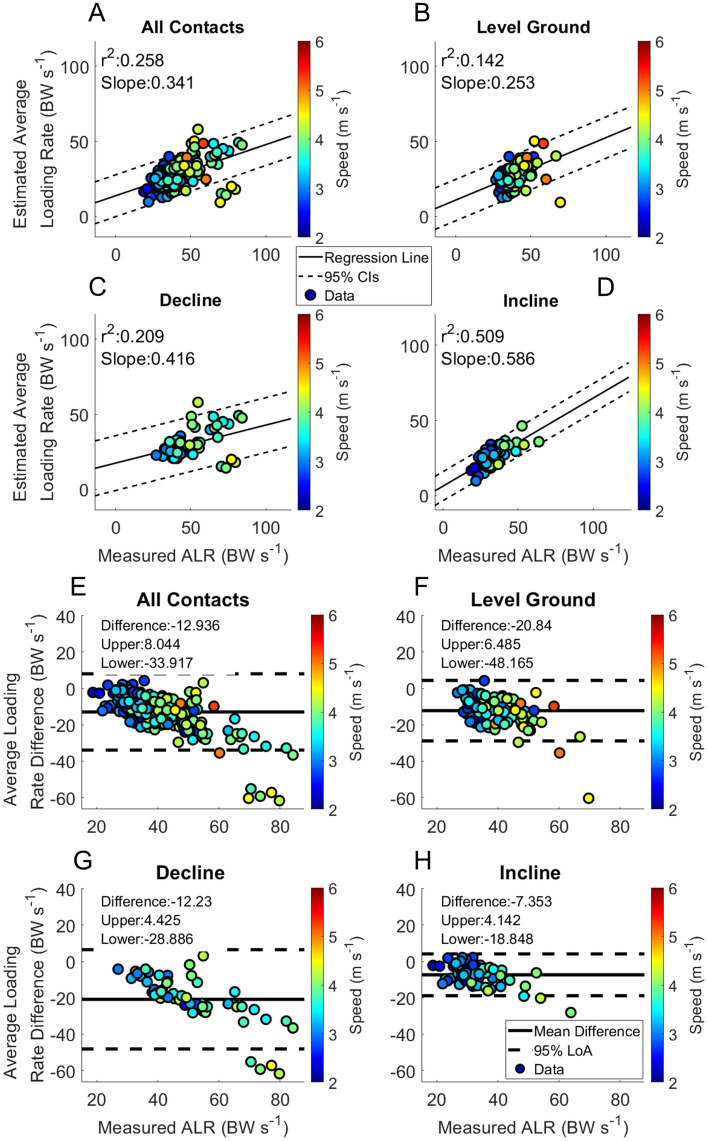
Average loading rate. Linear regression and Bland–Altman plots are presented for all foot contacts (**A** and **E**), followed by level ground (**B** and **F**), decline (**C** and **G**) and incline foot contacts (**D** and **H**). Pearson Correlation Coefficients and the slope of the regression line are presented in panels (**A**–**D**). The Bland–Altman plots present differences between the estimated and measured stance average GRF. The average difference and the 95% LoA are shown in panels (**E**–**H**).

Specific RMSEs for estimated temporal variables from the IMU heuristic algorithm and those estimated from the BD-LSTM across velocities and grades can be found Tables 2 and 3. Performance of foot contact estimation from the foot mounted IMU heuristic algorithm can be found in Figs. 1 and 2. Stride frequency as estimated from the force sensing insoles are presented in Fig. 4. Stance phase RMSE as estimated from the BD-LSTM is presented in Table 4. Kinetic variables measured from the force sensing insoles are presented in Table 5, and in Table 6 the RMSE from the BD-LSTM are presented. Kinetic variables across running velocity and slope are shown in Figs. 4 and 5. Estimated foot contact and kinetic variables from the BD-LSTM are presented in Figs. 6, 7, 8 and 9. Pearson correlation coefficients are presented as well as the slope of the regression line. Bland–Altman plots show mean difference in the estimated and measured variable with the 95% Limits of Agreement (LoA) (Figs. 2, 3, 6, 7, 8 and 9). Each data point in these figures represents a minimum of 10 footfalls for each velocity and grade (positive, negative and level ground) from the participant left out of the training set. These results show the range of the RMSE for calculated variables from both the heuristic and BD-LSTM algorithms across the range of speeds and slopes. Otherwise, if the data were directly measured from the IMUs or the force sensing insoles we present the minimum and the maximal values across the range of speeds and slopes.

**Table 2:** IMU heuristic temporal variable RMSE.

Running Speed (m s^−1^)	Initial contact			Toe off			Contact time		
	Level ground	Decline	Incline	Level ground	Decline	Incline	Level ground	Decline	Incline
2.25	0.018 ± 0.002	–	0.010 ± 0.000	0.034 ± 0.017	–	0.054 ± 0.000	0.032 ± 0.016	–	0.059 ± 0.000
2.50	0.017 ± 0.003	0.015 ± 0.006	0.016 ± 0.007	0.034 ± 0.017	0.046 ± 0.012	0.026 ± 0.009	0.032 ± 0.016	0.044 ± 0.015	0.020 ± 0.012
2.75	0.017 ± 0.003	0.019 ± 0.006	0.015 ± 0.006	0.028 ± 0.008	0.033 ± 0.013	0.022 ± 0.008	0.030 ± 0.011	0.032 ± 0.014	0.023 ± 0.009
3.00	0.019 ± 0.005	0.018 ± 0.006	0.017 ± 0.006	0.028 ± 0.009	0.034 ± 0.011	0.027 ± 0.009	0.027 ± 0.009	0.032 ± 0.007	0.027 ± 0.011
3.25	0.019 ± 0.005	0.018 ± 0.005	0.017 ± 0.005	0.027 ± 0.009	0.025 ± 0.009	0.024 ± 0.007	0.026 ± 0.009	0.024 ± 0.010	0.027 ± 0.010
3.50	0.018 ± 0.004	0.019 ± 0.007	0.017 ± 0.006	0.024 ± 0.006	0.028 ± 0.008	0.026 ± 0.008	0.025 ± 0.009	0.026 ± 0.010	0.026 ± 0.011
3.75	0.018 ± 0.004	0.019 ± 0.005	0.020 ± 0.005	0.025 ± 0.009	0.027 ± 0.012	0.025 ± 0.009	0.025 ± 0.011	0.027 ± 0.010	0.022 ± 0.010
4.00	0.020 ± 0.005	0.022 ± 0.011	0.020 ± 0.006	0.028 ± 0.011	0.025 ± 0.009	0.024 ± 0.011	0.028 ± 0.012	0.029 ± 0.011	0.021 ± 0.010
4.25	0.020 ± 0.007	0.017 ± 0.011	0.019 ± 0.007	0.028 ± 0.014	0.025 ± 0.011	0.020 ± 0.009	0.029 ± 0.012	0.025 ± 0.014	0.026 ± 0.011
4.50	0.019 ± 0.008	0.011 ± 0.007	–	0.026 ± 0.013	0.035 ± 0.008	–	0.033 ± 0.014	0.029 ± 0.012	–
4.75	0.018 ± 0.000	–	–	0.042 ± 0.000	–	–	0.036 ± 0.000	–	–
5.00	0.021 ± 0.000	0.017 ± 0.000	–	0.026 ± 0.025	0.010 ± 0.000	–	0.033 ± 0.022	0.024 ± 0.000	–
5.25	0.051 ± 0.000	–	–	0.045 ± 0.000	–	–	0.066 ± 0.000	–	–

**Table 3:** BD-LSTM temporal variable RMSE.

Running Speed (m s^−1^)	Initial contact			Toe off			Contact time		
	Level Ground	Decline	Incline	Level Ground	Decline	Incline	Level Ground	Decline	Incline
2.25	0.020 ± 0.014	–	0.023 ± 0.006	0.030 ± 0.019	–	0.025 ± 0.012	0.038 ± 0.022	–	0.025 ± 0.003
2.50	0.021 ± 0.006	0.039 ± 0.000	0.025 ± 0.008	0.030 ± 0.012	0.060 ± 0.000	0.030 ± 0.015	0.033 ± 0.017	0.047 ± 0.000	0.027 ± 0.006
2.75	0.020 ± 0.006	0.021 ± 0.014	0.019 ± 0.005	0.024 ± 0.010	0.023 ± 0.005	0.024 ± 0.009	0.028 ± 0.013	0.022 ± 0.005	0.027 ± 0.012
3.00	0.020 ± 0.006	0.019 ± 0.008	0.019 ± 0.005	0.022 ± 0.009	0.021 ± 0.006	0.022 ± 0.008	0.027 ± 0.014	0.024 ± 0.012	0.026 ± 0.011
3.25	0.020 ± 0.007	0.018 ± 0.008	0.019 ± 0.006	0.022 ± 0.009	0.025 ± 0.006	0.020 ± 0.007	0.027 ± 0.015	0.027 ± 0.012	0.024 ± 0.009
3.50	0.021 ± 0.007	0.021 ± 0.008	0.018 ± 0.006	0.023 ± 0.009	0.024 ± 0.009	0.021 ± 0.009	0.029 ± 0.016	0.027 ± 0.012	0.027 ± 0.011
3.75	0.021 ± 0.010	0.023 ± 0.011	0.016 ± 0.009	0.024 ± 0.009	0.029 ± 0.014	0.016 ± 0.005	0.030 ± 0.015	0.035 ± 0.017	0.021 ± 0.007
4.00	0.021 ± 0.009	0.024 ± 0.011	0.018 ± 0.010	0.026 ± 0.011	0.032 ± 0.011	0.019 ± 0.008	0.034 ± 0.016	0.039 ± 0.017	0.026 ± 0.009
4.25	0.021 ± 0.009	0.024 ± 0.010	0.023 ± 0.011	0.027 ± 0.013	0.030 ± 0.010	0.014 ± 0.002	0.034 ± 0.018	0.039 ± 0.017	0.021 ± 0.004
4.50	0.019 ± 0.005	0.022 ± 0.010	–	0.029 ± 0.007	0.043 ± 0.024	–	0.039 ± 0.011	0.051 ± 0.019	–
4.75	0.022 ± 0.000	–	–	0.026 ± 0.000	–	–	0.039 ± 0.000	–	–
5.00	0.025 ± 0.007	–	–	0.025 ± 0.009	–	–	0.040 ± 0.010	–	–
5.25	0.029 ± 0.000	–	–	0.036 ± 0.000	–	–	0.040 ± 0.000	–	–

**Table 4:** BD-LSTM stance phase ground reaction force waveform RMSE.

Running velocity (m s^−1^)	Level ground (BW)	Incline (BW)	Decline (BW)
2.25	0.315 ± 0.102	–	0.253 ± 0.081
2.50	0.313 ± 0.066	0.300 ± 0.000	0.265 ± 0.058
2.75	0.304 ± 0.057	0.332 ± 0.004	0.268 ± 0.067
3.00	0.294 ± 0.065	0.292 ± 0.082	0.272 ± 0.073
3.25	0.289 ± 0.059	0.321 ± 0.035	0.277 ± 0.065
3.50	0.299 ± 0.070	0.354 ± 0.070	0.274 ± 0.066
3.75	0.310 ± 0.072	0.389 ± 0.120	0.229 ± 0.036
4.00	0.339 ± 0.085	0.391 ± 0.165	0.309 ± 0.082
4.25	0.358 ± 0.115	0.394 ± 0.172	0.269 ± 0.007
4.50	0.397 ± 0.180	0.576 ± 0.371	–
4.75	0.376 ± 0.000	–	–
5.00	0.427 ± 0.010	–	–
5.25	0.635 ± 0.000	–	–

**Table 5:** Measured kinetic variables from force insoles (Mean ± SD).

Running speed (m s^−1^)	Stance average ground reaction force (BW)			Peak ground reaction force (BW)		
	Level ground	Decline	Incline	Level ground	Decline	Incline
2.25	1.350 ± 0.075	–	1.230 ± 0.021	2.318 ± 0.146	-	2.157 ± 0.052
2.50	1.314 ± 0.079	1.294 ± 0.000	1.230 ± 0.021	2.253 ± 0.152	2.168 ± 0.000	2.157 ± 0.052
2.75	1.327 ± 0.076	1.350 ± 0.084	1.277 ± 0.097	2.282 ± 0.165	2.249 ± 0.185	2.223 ± 0.203
3.00	1.294 ± 0.081	1.316 ± 0.085	1.257 ± 0.086	2.254 ± 0.151	2.248 ± 0.187	2.195 ± 0.163
3.25	1.291 ± 0.089	1.320 ± 0.066	1.253 ± 0.085	2.254 ± 0.154	2.279 ± 0.135	2.187 ± 0.151
3.50	1.314 ± 0.091	1.352 ± 0.118	1.264 ± 0.084	2.298 ± 0.162	2.338 ± 0.216	2.212 ± 0.146
3.75	1.317 ± 0.100	1.355 ± 0.133	1.280 ± 0.084	2.308 ± 0.186	2.367 ± 0.255	2.242 ± 0.156
4.00	1.337 ± 0.109	1.375 ± 0.141	1.278 ± 0.075	2.341 ± 0.206	2.401 ± 0.268	2.236 ± 0.130
4.25	1.338 ± 0.118	1.386 ± 0.178	1.305 ± 0.063	2.346 ± 0.230	2.431 ± 0.344	2.294 ± 0.128
4.50	1.335 ± 0.114	1.351 ± 0.245	–	2.353 ± 0.234	2.394 ± 0.435	–
4.75	1.457 ± 0.000	–	–	2.514 ± 0.000	–	–
5.00	1.515 ± 0.150	1.590 ± 0.000	–	2.669 ± 0.260	2.846 ± 0.000	–
5.25	1.449 ± 0.000	–	–	2.505 ± 0.000	–	–

	Impulse (BW*s)			Average loading rate (BW s^−1^)		
	Level Ground	Decline	Incline	Level Ground	Decline	Incline
2.25	0.356 ± 0.023	–	0.365 ± 0.024	40.380 ± 8.429	–	26.897 ± 4.971
2.50	0.373 ± 0.031	0.354 ± 0.000	0.365 ± 0.024	31.919 ± 1.564	46.045 ± 0.000	26.897 ± 4.971
2.75	0.369 ± 0.026	0.365 ± 0.024	0.370 ± 0.024	35.168 ± 5.992	50.658 ± 11.145	29.969 ± 4.459
3.00	0.357 ± 0.029	0.366 ± 0.026	0.361 ± 0.029	34.657 ± 5.588	38.220 ± 6.578	30.215 ± 3.581
3.25	0.352 ± 0.027	0.353 ± 0.024	0.354 ± 0.028	36.008 ± 6.455	45.744 ± 12.826	30.755 ± 4.304
3.50	0.350 ± 0.028	0.357 ± 0.028	0.353 ± 0.027	39.088 ± 5.242	47.238 ± 13.109	32.381 ± 4.213
3.75	0.346 ± 0.029	0.348 ± 0.029	0.348 ± 0.027	39.984 ± 5.073	51.401 ± 10.231	36.196 ± 4.405
4.00	0.348 ± 0.030	0.347 ± 0.029	0.342 ± 0.026	43.588 ± 5.613	55.461 ± 9.529	38.833 ± 4.716
4.25	0.346 ± 0.032	0.347 ± 0.036	0.342 ± 0.026	46.055 ± 8.777	55.107 ± 11.352	43.496 ± 8.523
4.50	0.342 ± 0.032	0.340 ± 0.041	–	47.118 ± 6.174	47.399 ± 17.226	–
4.75	0.398 ± 0.000	–	–	40.092 ± 0.000	–	–
5.00	0.374 ± 0.042	0.403 ± 0.000	–	57.365 ± 14.135	57.359 ± 0.000	–
5.25	0.333 ± 0.000	–	–	58.307 ± 0.000	–	–

**Table 6:** Kinetic variable RMSE from BD-LSTM estimated waveforms.

Running speed (m s^−1^)	Stance average ground reaction Force (BW)			Peak ground reaction force (BW)		
	Level Ground	Decline	Incline	Level Ground	Decline	Incline
2.25	0.160 ± 0.068	–	0.089 ± 0.013	0.222 ± 0.087	–	0.117 ± 0.042
2.50	0.149 ± 0.063	0.191 ± 0.000	0.106 ± 0.021	0.233 ± 0.097	0.430 ± 0.000	0.115 ± 0.021
2.75	0.153 ± 0.047	0.132 ± 0.003	0.122 ± 0.048	0.201 ± 0.091	0.218 ± 0.042	0.182 ± 0.106
3.00	0.134 ± 0.055	0.123 ± 0.061	0.121 ± 0.049	0.187 ± 0.097	0.197 ± 0.128	0.169 ± 0.102
3.25	0.141 ± 0.058	0.138 ± 0.068	0.118 ± 0.044	0.194 ± 0.097	0.205 ± 0.109	0.154 ± 0.089
3.50	0.153 ± 0.070	0.185 ± 0.061	0.124 ± 0.056	0.208 ± 0.114	0.268 ± 0.126	0.163 ± 0.119
3.75	0.162 ± 0.079	0.227 ± 0.126	0.106 ± 0.023	0.220 ± 0.129	0.351 ± 0.194	0.138 ± 0.038
4.00	0.186 ± 0.088	0.212 ± 0.151	0.158 ± 0.059	0.255 ± 0.132	0.316 ± 0.241	0.213 ± 0.110
4.25	0.204 ± 0.100	0.233 ± 0.159	0.139 ± 0.017	0.259 ± 0.155	0.345 ± 0.251	0.136 ± 0.031
4.50	0.245 ± 0.171	0.337 ± 0.315	–	0.311 ± 0.261	0.508 ± 0.416	–
4.75	0.247 ± 0.000	–	–	0.392 ± 0.000	–	–
5.00	0.298 ± 0.076	–	–	0.380 ± 0.252	–	–
5.25	0.313 ± 0.000	–	–	0.223 ± 0.000	–	–

	Impulse (BW*s)			Average loading rate (BW s^−1^)		
	Level Ground	Decline	Incline	Level Ground	Decline	Incline
2.25	0.024 ± 0.004	–	0.022 ± 0.010	19.208 ± 4.419	–	13.043 ± 2.744
2.50	0.038 ± 0.015	0.086 ± 0.000	0.024 ± 0.011	14.963 ± 4.868	28.065 ± 0.000	12.242 ± 4.931
2.75	0.035 ± 0.008	0.036 ± 0.006	0.030 ± 0.012	15.399 ± 4.307	24.423 ± 5.967	12.915 ± 3.642
3.00	0.031 ± 0.011	0.035 ± 0.011	0.030 ± 0.014	16.549 ± 4.926	18.346 ± 6.540	13.225 ± 3.691
3.25	0.031 ± 0.011	0.036 ± 0.013	0.029 ± 0.015	18.065 ± 4.870	22.735 ± 4.258	15.267 ± 4.550
3.50	0.031 ± 0.009	0.040 ± 0.011	0.031 ± 0.011	18.830 ± 4.966	27.728 ± 5.564	15.812 ± 4.568
3.75	0.034 ± 0.010	0.043 ± 0.011	0.032 ± 0.008	20.028 ± 4.578	30.510 ± 2.926	13.566 ± 2.308
4.00	0.039 ± 0.012	0.037 ± 0.013	0.039 ± 0.014	22.185 ± 5.708	32.335 ± 13.041	19.349 ± 9.038
4.25	0.039 ± 0.014	0.043 ± 0.025	0.035 ± 0.009	25.270 ± 5.889	32.324 ± 13.928	21.642 ± 3.445
4.50	0.047 ± 0.018	0.047 ± 0.018	–	30.374 ± 19.786	45.121 ± 18.762	–
4.75	0.058 ± 0.000	–	–	31.505 ± 0.000	–	–
5.00	0.066 ± 0.009	–	–	33.656 ± 9.727	–	–
5.25	0.058 ± 0.000	–	–	34.680 ± 0.000	–	–

### IMU heuristic gait event and contact time

The average RMSE across speeds and slopes of the estimated initial contact from the IMU heuristic (IC_IMU_) are presented in Table 2. Differences between the IC_IMU_ and the measured initial contact (IC) are shown in (Fig. 1 Panel A). Estimation of toe off from the IMU heuristic (TO_IMU_) are presented in Table 2. Differences between the TO_IMU_ and the measured toe off (TO) are shown in Fig. 2 Panel B. The RMSE across speeds of estimated contact times are presented in (Table 2). Estimated contact times from the IMU are presented in Fig. 1 Panel C, and the measured foot contact times from the force sensing insoles are presented in Fig. 1 Panel D. Linear regression and the analysis of bias in the estimate can be found in Fig. 2.

### Variables calculated from force sensing insoles

Stride frequency was observed to change across velocities but minimally with slope. Stride frequencies are displayed in (Fig. 4). Measured stance average GRFs are presented in Fig. 5 Panel A. Peak GRFs are presented in Fig. 5 Panel B. Impulse during stance phase across speeds are presented in Fig. 5 Panel C. Measured ALR across speeds is presented in Fig. 5 Panel D.

### BD-LSTM temporal and kinetic variables

The RMSE of the estimation across speeds of Initial Contact from the BD-LSTM (IC_LSTM_) are shown in Table 3. Estimation RMSE across speeds of Toe Off from the BD-LSTM (TO_LSTM_) are presented in Table 3. Contact time estimation RMSE across speeds; Table 3. Linear regression analysis and Bland Altman plots from the BD-LSTM are presented in Fig. 3. Stance phase GRF whole waveform RMSE across speeds ranged from 0.30 BW to 0.64 BW across all running velocities and grades Table 4. Stance average GRF RMSE are stated in Table 5. Peak force RMSE are displayed in Table 5. Impulse RMSE are presented in Table 5. Average loading rate RMSE are presented in Table 5. Figures 6, 7, 8 and 9 present linear regression analysis and Bland Altman plots for the estimated GRF variables.

## Discussion

The purpose of this study was to test two specific methods for the biomechanical analysis of running in an unconstrained environment: (1) a heuristic algorithm for the estimation of foot contacts from IMU data; (2) a machine learning algorithm, BD-LSTM, for estimation of normal GRFs between the foot and shoe, specifically foot contact events and discrete GRF variables. The specific findings of the study are summarized here: (1) contact time with foot-mounted IMUs was estimated with an average RMSE of 0.030 s, (2) BD-LSTM output waveforms estimated contact times with RMSE of 0.031 s, (3) BD-LSTM output waveform step-by-step average for all combinations of velocities and grades had an RMSE of 0.33 BW per step. Throughout the discussion, it was assumed the greater ranges of RMSEs, lower Pearson Correlation Coefficients and wider 95% LoA are due to three potential sources of error, (1) the unconstrained running environment of this study in comparison to running in a controlled laboratory environment. (2) the lack of feature engineering as the process presented is completely data driven. (3) The data presented are across a 13-fold LOOCV, data such as this have not yet been presented, as normally representative participants have presented in past work.

### Validation of ground reaction force variables

We observed a decrease in estimated contact time with increased running velocity, for level ground, incline and decline foot contacts (Fig. 1 Panel C). Minimal differences were noted in measured contact times between level ground, incline, and decline (Fig. 1 Panel D). Comparison of stride frequencies for running velocities from 2.5 to 4.5 m s^−1^ between the current study and a treadmill study showed minimal differences ranged from [−  2.60 − 4.64] strides min^−1^^31^. There were negligible differences between level ground running, decline and incline stride frequencies (Fig. 4). This finding is not surprising, as velocity has been shown to have a larger effect on stride frequency^32,33^. However, we have shown that stance average GRFs, peak GRFs and ALR increased with running velocity (Fig. 5), following the same trends previously reported^31,34^. The current study measured impulses ranging from 0.33 to 0.40 BW*s (Table 5), compared to another study that reported impulse across different velocities and grades on a treadmill ranging from 0.30 to 0.34 BW*s^22^. The range of ALR in our study (31.92–58.31 BW s^−1^) is similar to previous work (30.10–64.70 BW s^−1^)^22^ across a variety of velocities and grades. Differences in ALR during decline running showed an increase of 9.3 BW s^−1^, and decrease of 2.05 BW s^−1^ during incline running (Fig. 5), which is similar to values reported previously^33^.

### Estimation of gait events and foot contacts with IMUs and BD-LSTM

Our approach to estimating gait events used both acceleration and angular velocity data, which differs from previous work, as most studies have made use of only one type of data, either accelerations or angular velocities^5,8,35–40^. Differences between IC_IMU_ and measured IC in the current study occurred in the expected range (− 0.020–0.020 s), due to the iterative corrections algorithm used. A previous study in a controlled laboratory environment reported identification of IC_IMU_ across a small range of velocities (8–11 km h^−1^), with a range of RMSE 0.004–0.008 s^35^. This is a smaller average RMSE than the current study, with the current IC_IMU_ RMSE range 0.011–0.051 s. The same previous study reported a larger RMSE range for identification of TO_IMU_: 0.008–0.011 s, while our study presented a TO_IMU_ RMSE range from 0.020 to 0.053 s^35^. Machine learning estimation of gait events allows for flexibility in the identification of IC_LSTM_ and TO_LSTM_ instead of relying on specific heuristics, as presented above. We have shown minimal differences between the IMU heuristic estimated contact time and the BD-LSTM estimated contact time; RMSE ranges for IC_IMU_ of 0.011–0.051 s, compared to a range of 0.016–0.039 s for IC_LSTM_. There was a larger RMSE in the lower bound of IC_LSTM_, but a narrower range of RMSEs across the range of running velocities. Estimation of TO_IMU_ had an RMSE range of 0.020–0.053 s, while TO_LSTM_ RMSE was 0.014–0.059 s (Tables 2 and 3). The estimation of TO with inertial sensors has shown more variability than estimation of IC in many different studies, including the current study^5,9,35,36^.

Contact time estimated from both the foot mounted IMUs and the BD-LSTM decreased with increased running velocity (Figs. 2 and 3). Contact time estimation calculated from the heuristic algorithm in this study had an RMSE from 0.020 to 0.066 s. Contact time estimated from the BD-LSTM had an RMSE that ranged from 0.021 to 0.040 s, an improvement over the heuristic estimated contact time. Foot contact durations for IMU estimates had an r^2^ = 0.460, and the BD-LSTM estimated foot contact durations had an r^2^ = 0.524 (Figs. 2 Panel E, and Fig. 3 Panel E). Despite better agreement in the output from the BD-LSTM, there was more bias in the estimation of contact time from the BD-LSTM compared to the heuristic calculated contact time, (Level Ground: 0.010 s vs. 0.005 s), and this trend continued with the different grade conditions (Figs. 2 and 3 Panels E–F). Another study reported an r^2^ = 0.665 for estimated contact times from a Quantile Regression Forest, while the current study presented an r^2^ = 0.524 across all foot contacts ^14^. For external comparison, our model showed a reduced bias in the estimation of contact time compared to Benson et al.^5^. They reported an offset of − 0.016 s with 95% LoA [− 0.058 0.027 s], while the current model resulted in an offset of 0.005 s with 95% LoA [− 0.035 0.044 s] across all heuristic calculated foot contacts, and an offset of 0.010 s with 95% LoA [− 0.025 0.044 s] across all BD-LSTM estimated foot contacts. Our approach resulted in narrower limits of agreement for both the heuristic and BD-LSTM estimated contact times. The previous study used raw data for analysis, compared to the use of averages of each combination of velocity and grade presented in this work.

### BD-LSTM ground reaction force analysis

Accuracy in the estimation of GRF waveforms during stance phase using the BD-LSTM varied across running velocities (Table 4). Level ground running had the largest RMSE range (0.29–0.64 BW), compared to decline running (0.29–0.58 BW) and incline running (0.23–0.31 BW). It should be noted however that level ground running also had the largest range of velocities (Table 3). We observed that the BD-LSTM underestimated GRF across the full range of velocities and grades. Stance phase RMSE ranged from 0.23 BW to 0.64 BW for all velocities and grades, compared to a previously estimated stance phase RMSE ranging from 0.12 to 0.20 BW derived from kinetic waveforms estimated from a machine learning algorithm for treadmill running at different velocities and inclinations^22^.

Stance average forces mirrored the estimation of the whole waveform. Correlation of stance average GRFs reported in previous work from our group was r^2^ = 0.408 across running velocities^41^. However, the current analysis yielded a much lower agreement; r^2^ = 0.105. The current study had 95% LoA [− 0.33 0.11] BW and a mean difference of − 0.11 BW for all foot contacts (Fig. 6). This is slightly more bias than reported in previous work from our group (mean difference = − 0.09 BW)^41^. For external comparison, an LSTM was developed for the estimation of stance average GRFs reported an RMSE between 0.34 and 0.63 BW^15^, while the current study reported a stance average GRF RMSE between 0.09 and 0.31 BW.

Estimated peak force had similar patterns to the estimated stance average GRF (Fig. 7). The correlation of peak force reported in previous work from our laboratory was r^2^ = 0.614 for level ground steady state running velocities, while the current work resulted in an r^2^ = 0.332 across all foot contacts, with worse performance in the estimation of peak force during incline running foot contacts (r^2^ = 0.264)^41^. Previous work reported a moderate correlation between the estimated and measured peak GRFs, from data collected on a force instrumented treadmill (r^2^ = 0.665)^14^. For further external comparison, a BD-LSTM that utilized only foot contact information from a single sensor on the sacrum resulted in an r^2^ = 0.62, with 95% LoA [− 0.17 0.18] BW and a bias of 0.01 BW. In another study, the 95% LoA ranged from [− 0.50 0.22] BW with a bias of − 0.14 BW^15^. The major difference between the previous work and ours was that they estimated single stance phase vertical GRFs on a treadmill, while we estimated entire waveforms in a free running environment, which is inherently more variable than in the controlled laboratory setting.

Impulse had the best performance of the calculated discrete kinetic variables from the estimated GRF waveform, as it was the least effected by underestimation of the force waveform magnitude. Linear regression showed that estimated and measured impulse were moderately correlated, r^2^ = 0.571 for all foot contacts (Fig. 8 Panel A). Impulse was underestimated by 0.04 BW*s for all foot contacts, which equates to approximately 6–8% error in the estimation of impulse across the range of locomotion velocities and grades. More precise estimation of contact time from the BD-LSTM increased the impulse calculation accuracy. In comparison to previous work from our laboratory, estimated impulse was weakly correlated with the measured impulse, r^2^ = 0.385, with a bias of 0.01 BW*s and 95% LoA [− 0.05 0.07] BW*s, while for the current study we observed an r^2^ = 0.571, a bias of − 0.02 BW*s and 95% LoA [− 0.06 0.02] BW*s^41^. A different group reported estimated impulse with a mean absolute error of approximately 0.03 BW*s across velocities and grades running on a treadmill^22^, compared to the current study with RMSE across running velocities and grades ranging from 0.02 to 0.09 BW*s. The RMSEs for impulse in the present study tended to be larger for decline running compared to level ground and incline running, due to the more pronounced impact peak observed in that condition.

Estimated ALR was weakly correlated with measured ALR, with r^2^ = 0.160 for level ground running, r^2^ = 0.210 for decline running, and r^2^ = 0.492 for incline running. In a previous study by our group, correlation between the estimated and measured ALR during level ground running on a track surface yielded an r^2^ = 0.614^41^. For comparison, in another study using data collected in a laboratory environment across a range of velocities, loading rate was moderately correlated to measured loading rate, with an r^2^ = 0.57, a bias of − 2.9 BW s^−1^ 95% and LoA [− 16 10] BW s^−1^^15^, while the current study presents a 95% LoA [34.00 7.92] BW s^−1^ with a bias of − 13.04 BW s^−1^. Other work has presented a direct estimation of Vertical Average Loading Rate (VALR), in which the performance of their model at two running velocities and in the laboratory environment had a correlation coefficient of 0.93. The output of this model was specifically VALR, and utilized IMUs and data from force plates in a highly controlled environment^42^. Estimated ALR has been reported to have larger percent errors than other estimated kinetic variables^22^. In the present study, the errors in our estimation may be due to the inherent differences in loading rate between decline, incline, and level running and the unconstrained environment. The ALR is typically much larger for decline running than it is for incline or level ground running^33^.

There are various limitations in the collection of running data outside of the laboratory, some of which have been highlighted above. There were two corrections made to the force data in this study. The first of these was an iterative corrections algorithm to resolve differences between the internal clocks of the IMU and the force sensing insoles. Second, throughout the study, approximately 500 footfalls were observed to have a drifting baseline. The drifting baseline may have been a result of the force sensing insole moving between the foot and the shoe during the highly dynamic running activities being tested. For example, the ALR may have reduced accuracy due to the low sampling rate of 100 Hz, compared to in lab studies where the force plate data are sampled at > 1000 Hz^22,42^. Improvements in wearable sensors, such as increased signal sampling rates and fidelities would likely improve the outcomes of future work in this domain. There was also a small error in the synchronization of GPS data to IMU and GRF. This protocol greatly improved our data analysis capacity, but unfortunately there remained a slight offset in the GPS data for each footfall. This can be rectified by the use of on-board GPS with the IMUs or force sensing insoles, which will lead the to the GPS being hard synced. The integration of these sensor networks would provide improved synchronization and lead to fewer assumptions in the methodology.

The BD-LSTM algorithm shows promise for machine learning paradigms to estimate running GRF waveforms. However, the current algorithm appears to have limited transferability to a novel participant, as evidenced by the inferior performance in the estimation of kinetic variables, especially at faster running velocities. There may have been significant over training of the model for level ground running speeds, therefore in the future it may be necessary to have different models for different slopes, velocities, and terrain. Performance would likely improve with the addition of more data at faster running velocities, and the inclusion of more participants to introduce more variability into the dataset. Additions to the neural network architecture and training data are necessary to improve model output before these algorithms are ready for application in the clinical or research setting. These may include the inclusion of features extracted from the waveform, or other data such as stride frequency, velocity and slope. In future work, it may be necessary for the use of feature engineering in addition to the use of temporal windowing for estimation of GRFs while running, as the model currently over fit to the mean of the level ground running ground reactions forces.

In conclusion, this is the first study to our knowledge to report kinetic measures during free run outside of the laboratory, despite having inherent limitations in its transferability to biomechanical analysis of running in the real world. The purpose of this paper was to implement a fully data driven technique to estimate ground reaction forces from data collected from persons running in a real-world environment. While the results an applicability of this study are limited, it does highlight the potential of these algorithms as the LOOCV results are presented, when data from a given participant is included the error in the model is expected to be significantly reduced. Further, this is the first study to estimate IMU contact times and validate them against a kinetic standard with data collected in a real-world running environment across a wide range of running velocities and slopes. We have also shown that the BD-LSTM architecture can be used to estimate kinetic waveforms via machine learning from running data collected in the real world, without feature engineering. We have shown estimation of gait events and contact time using IMU data matches the estimation of the same variables from a machine learning algorithm. Future studies focusing on building models for training load, single participant machine learning models, and direct inclusion of GPS data as input may reduce the underestimation of the stance phase GRFs at faster running velocities.

## Methods

This study was approved by the University of Oregon Institutional Review Board (protocol #: 10062020.007). All participants provided written informed consent prior to enrolling in the study. All research procedures adhered to the principles defined in the Declaration of Helsinki. Data were collected from 16 participants (Table 1), (8 male, 8 female, age: 23.2 years, height: 167.8 cm, mass: 65.0 kg) as part of a larger ongoing study. Three participants were excluded from the analysis, due to GPS malfunctions. All analyses were performed in custom Matlab programs (MathWorks, Natick, MA, USA)^48^. Multi-axial IMUs (Casio Computer Co., LTD, Tokyo, Japan) were mounted bilaterally on the dorsal aspect of each participant’s foot and approximately on the sacrum (clipped on the back of each participant’s waistband). Each of the IMUs were oriented such that the x-axis of the IMU was in alignment with the sagittal plane. The use of multiple inertial sensors has been suggested to improve estimation of spatial temporal and kinetic variables, compared to a single inertial sensor^10,43,44^. These multi-axial IMUs recorded 3D linear accelerations and angular velocities at 200 Hz. Acceleration data were post-processed with a Kalman filter to orient the local (IMU) coordinate system vertical to gravity. Foot-shoe normal force data were recorded with Loadsol insole force sensors (Novel Electronics, St. Paul, MN, USA) at 100 Hz. Participants were asked to run a five-mile course around the University of Oregon and surrounding parks. Participants also wore a GPS watch (Forerunner 130 or 135; Garmin, Kansas City, KS, USA) to record elevation and running velocity. These data were exported to Garmin Connect (https://www.garminconnect.com/) and then second by second running velocity and percent grade were extracted with Golden Cheetah v3.5 (https://www.goldencheetah.org/).

## Data processing

Force-sensing insole and IMU data were synced with ‘foot stomps’ before and after each run. The IMU data were downsampled to 100 Hz to match the force sensing insoles and filtered with a 4th order low pass zero-lag Butterworth filter (*fc* = 35 Hz). Internal clock drift between the IMUs and force sensing insoles was resolved with an iterative corrections algorithm. The kinetic data were normalized to each participant’s bodyweight (BW) and were filtered with a 4th order low-pass zero-lag Butterworth filter (*fc* = 20 Hz). Post-hoc corrections to force insole data due to a drifting baseline were made as needed. Making these corrections entailed identifying swing phases during a period in which the forces had a drifting baseline and setting the swing phases to 0 BW. Less than 1% of the measured footfalls needed this adjustment. Additionally, force data < 5% BW were set to 0 BW.

Synchronization of IMU and force data to the GPS was achieved by matching the sudden increase in velocity measured by the GPS to the beginning of the run, and the periods in which the runner had minimal velocity (e.g., while waiting at street crossings). Elevation and velocity measured by the GPS (*sf* = 1 Hz) were filtered with a zero-lag 10-sample moving average filter. Velocities from GPS data were set to the nearest 0.25 m s^−1^ ranging from 2.25 to 5.25 m s^−1^, and the upper limit for running velocity was set by the number of footfalls available for analysis. Running velocities < 2.25 m s^−1^ are typically walking velocities and the walk to run transition typically occurs at around 2.00–2.10 m s^−1^^45^. Grade was calculated from the elevation data and binned into three different groupings. Incline foot strikes were identified at measured grades of > 5, and decline foot strikes were identified as measured grades of < − 5°, with level ground foot strikes between 5° and − 5°. The range of grades that were considered level ground running [− 5°, 5°] was set due to observed noise of ± 4° throughout the run during portions of the course with no physically discernible grade. Data from the GPS were then time-synced to the IMU and kinetic data. For data to be included in the analysis, a minimum of 10 footfalls for a combination of velocity and grade were required from a given participant. From the force sensing insoles, we calculated stride frequency, stance average GRF, peak GRF, impulse and average loading rate (ALR). Average loading rate was calculated by identifying the impact peak and then calculating the force/time slope in the middle 60% of the region between initial contact (IC) and the impact peak^46^.

### Gait event detection algorithms

Gait event estimation, initial contact (IC) and toe off (TO) from IMU data utilized heuristic rules similar to previous work^5,8,36^. Initial contact from the IMUs on the dorsal aspect of the foot (IC_IMU_) was identified with two rules. First was the identification of minimum angular velocity about the x-axis of the IMU with a minimum of 0.500 s between identified minima. Second, a temporal window relative to each minimum, ranging from 0.005 s to 0.045 s post was searched for a resultant acceleration > 50 m s^−2^. If this condition was satisfied then the peak resultant acceleration was set to be IC_IMU_^5,35,36^. Identification of Toe off from the IMU (TO_IMU_) was performed by searching a specific temporal window beginning 0.010 s after IC_IMU_ and ending at the half-width of the estimated stride time. In this window TO_IMU_ was either identified as the local maxima of vertical acceleration or the first instance that vertical acceleration was > 3* g*^5,47^. Identification of gait events with foot-shoe normal force data utilized a 5% BW cutoff; the first instance of force > 5% BW was identified as IC, and TO was identified as the last instance of force < 5% BW. We then removed foot contacts that could not be matched to the IMU and force sensing insole measures. If IC_IMU_ was not within half contact time of the IC from the force sensing insole it was removed from the analysis.

### Machine learning architecture and analysis

**Figure 10:**
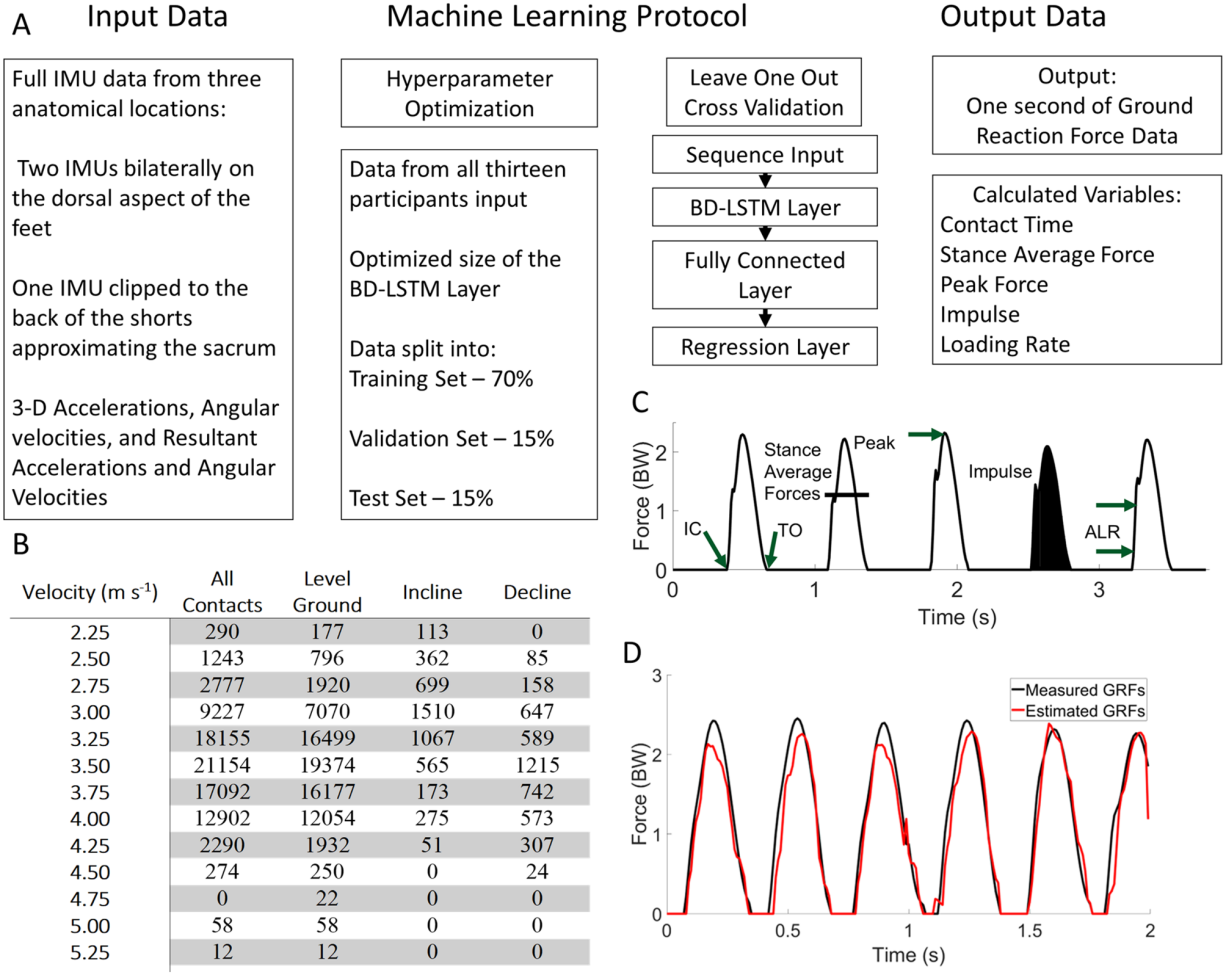
Outline of the machine learning protocol in Panel (**A**). The number of steps in each velocity and grade are presented in Panel (**B**). In panel (**C**), the temporal and kinetic variables calculated from both the measured and estimated waveforms. Panel (**D**) shows data from two separate one second windows which were concatenated.

The hyperparameters of the BD-LSTM were optimized with a Bayesian Hyperparameter Optimization algorithm from Matlab^48^. The only hyperparameter found to have a significant effect on the model was the number of hidden units in the LSTM layer, which was optimized at 19 from a range of 10 to 1000 hidden units. All other hyperparameters were set to the Matlab defaults. The temporal windowing was identified with a sweep of window lengths ranging from one second to five seconds at half second intervals. One second window lengths were found to have the most accurate results with each of the networks from the Bayesian optimization process. Using the ADAM algorithm, the learning rates were initialized at the Matlab default of 0.001. The model consisted of a sequence input layer, the LSTM layer (standard activations at each of the gates), a fully connected layer (sigmoid activation) and a regression layer. The number of footfalls held out from the LOOCV are shown in the steps measured column of Table 1. The workflow for the hyperparameter optimization is shown in (Fig. 10 Panel A). The loss function for the BD-LSTM was mean squared error.

We utilized a BD-LSTM with 19 hidden units and a regression output. A more thorough description of the network architecture can be found here^25^. The activations of the BD-LSTM were the standard LSTM activation functions, and the regression layer had a linear activation function. The number of epochs was 100, and the batch size was 50. Input into the BD-LSTM were 1-s windows of inertial data: 3-D accelerations, angular velocities, and their respective resultants, from three anatomical locations (dorsum of both feet, and the waistband at approximately the sacrum). Output from the BD-LSTM were 1-s intervals of estimated GRF data, the summation of the GRF waveforms from both force sensing insoles. The algorithm was evaluated with a Leave One Out Cross Validation (LOOCV) with 12 participants in the training data and 1 participant in the test data, repeated for each participant. The estimated force data were then filtered with a 2nd order low-pass zero-lag Butterworth filter (*fc* = 15 Hz). Estimated data tended to be noisier than the input GRF waveforms. This was accounted for with a lower cutoff frequency in the filter (Fig. 10 Panel D). Errant estimated GRF data were removed by setting estimated force < 5% BW to 0 BW, and by removal of false “foot-contacts” generated by the model that were < 0.100 s or > 0.500 s. Foot contacts shorter than 0.100 s were not consistent with measured foot contacts during running and foot contacts longer than 0.500 s tended to occur during periods of quiet standing (e.g. participant was at a street crossing). We observed that the swing phase estimation error approached 0 as most of the errant data were corrected for using the steps described above. Initial contact from the machine learning output (IC_LSTM_) was identified by the first instance of force > 5% BW and toe off (TO_LSTM_) was identified by the last instance of force greater than > 5% BW. To ensure matching foot contact correctly during analysis, if IC_LSTM_ was not within a half contact time of the measured IC, it was removed from the analysis. The total number of footfalls analyzed per speed are shown in (Fig. 10 Panel B). From the model output GRF waveforms, stance average GRFs, peak GRFs, impulse and ALR were calculated (Fig. 10 Panel C).

Statistical analysis included RMSE, linear models and bias analyses to assess estimated contact time, calculated as the temporal difference between TO and IC as measured by the force sensing insole, and the kinetic variables. Differences between the model estimated variable and measured variable waveform are presented in both linear regression and Bland–Altman plots with 95% confidence intervals (CIs) or Limits of Agreement (LoA), respectively. Pearson correlation coefficients (r^2^) were calculated to show agreement between estimated and measured data. A strong correlation was defined as r^2^ ≥ 0.8, a moderate correlation as 0.5 ≤ r^2^ ≤ 0.8 and a weak correlation as 0.3 ≤ r^2^ ≤ 0.5.

## Data Availability

The datasets used and/or analyzed during the current study available from the corresponding author on reasonable request.
